# Integrating Academic and Community Cancer Care and Research through Multidisciplinary Oncology Pathways for Value-Based Care: A Review and the City of Hope Experience

**DOI:** 10.3390/jcm10020188

**Published:** 2021-01-07

**Authors:** Linda D. Bosserman, Mary Cianfrocca, Bertram Yuh, Christina Yeon, Helen Chen, Stephen Sentovich, Amy Polverini, Finly Zachariah, Debbie Deaville, Ashley B. Lee, Mina S. Sedrak, Elisabeth King, Stacy Gray, Denise Morse, Scott Glaser, Geetika Bhatt, Camille Adeimy, TingTing Tan, Joseph Chao, Arin Nam, Isaac B. Paz, Laura Kruper, Poornima Rao, Karen Sokolov, Prakash Kulkarni, Ravi Salgia, Jonathan Yamzon, Deron Johnson

**Affiliations:** 1 Department of Medical Oncology and Therapeutics Research, City of Hope, Duarte, CA 91010, USA; mcianfrocca@coh.org (M.C.); msedrak@coh.org (M.S.S.); jchao@coh.org (J.C.); anam@coh.org (A.N.); pkulkarni@coh.org (P.K.); rsalgia@coh.org (R.S.); 2 Department of Surgery, City of Hope, Duarte, CA 91010, USA; byuh@coh.org (B.Y.); ssentovich@coh.org (S.S.); lkruper@coh.org (L.K.); jyamzon@coh.org (J.Y.); 3 Department of Medical Oncology and Therapeutics Research, City of Hope, South Pasadena, CA 91030, USA; cyeon@coh.org; 4 Department of Radiation Oncology, City of Hope, South Pasadena, CA 91030, USA; hechen@coh.org; 5 Department of Surgery, City of Hope, South Pasadena, CA 91030, USA; apolverini@coh.org (A.P.); bpaz@coh.org (I.B.P.); porao@coh.org (P.R.); 6 Department Supportive Care Medicine, City of Hope, Duarte, CA 91010, USA; fzachariah@coh.org; 7 Department of Enterprise Business Intelligence, City of Hope, Duarte, CA 91010, USA; ddeaville@coh.org; 8 Department of Clinical Research Operations, City of Hope, Duarte, CA 91010, USA; ashlee@coh.org; 9 Department of Population Sciences in the Division of Clinical Cancer Genomics, City of Hope, Duarte, CA 91010, USA; eking@coh.org (E.K.); stagray@coh.org (S.G.); 10 Department of Quality, Risk and Regulatory Management, City of Hope, Duarte, CA 91010, USA; dmorse@coh.org; 11 Department of Radiation Oncology, City of Hope, Duarte, CA 91010, USA; sglaser@coh.org; 12 Department of Medical Oncology and Therapeutics Research, City of Hope, Lancaster, CA 93534, USA; gbhatt@coh.org; 13 Department of Medical Oncology and Therapeutics Research, City of Hope, Upland, CA 91784, USA; cadeimy@coh.org; 14 Department of Medical Oncology and Therapeutics Research, City of Hope, Newport Beach, CA 92660, USA; titan@coh.org; 15 Department of Radiation Oncology, City of Hope, Torrance, CA 90503, USA; ksokolov@coh.org; 16 Department of Clinical Informatics, City of Hope, Duarte, CA 91010, USA; derjohnson@coh.org

**Keywords:** value-based care, value-based cancer care, oncology pathways, Early Recovery After Surgery (ERAS), team-based care, oncology medical home, integrated cancer care, supportive care pathways, surgical pathways, cancer care plans

## Abstract

As the US transitions from volume- to value-based cancer care, many cancer centers and community groups have joined to share resources to deliver measurable, high-quality cancer care and clinical research with the associated high patient satisfaction, provider satisfaction, and practice health at optimal costs that are the hallmarks of value-based care. Multidisciplinary oncology care pathways are essential components of value-based care and their payment metrics. Oncology pathways are evidence-based, standardized but personalizable care plans to guide cancer care. Pathways have been developed and studied for the major medical, surgical, radiation, and supportive oncology disciplines to support decision-making, streamline care, and optimize outcomes. Implementing multidisciplinary oncology pathways can facilitate comprehensive care plans for each cancer patient throughout their cancer journey and across large multisite delivery systems. Outcomes from the delivered pathway-based care can then be evaluated against individual and population benchmarks. The complexity of adoption, implementation, and assessment of multidisciplinary oncology pathways, however, presents many challenges. We review the development and components of value-based cancer care and detail City of Hope’s (COH) academic and community-team-based approaches for implementing multidisciplinary pathways. We also describe supportive components with available results towards enterprise-wide value-based care delivery.

## 1. Introduction: Multidisciplinary Oncology Pathways Are a Foundation of Value-Based Cancer Care

While the majority of cancer care in the US is provided in the community (non-academic) setting, over the past 12 years, the organizational structure of oncology care delivery has shifted to networks of community oncologists partnered with academic centers, hospital systems, or other community practices. Aligned enterprises have evolved from the well-documented closing, merging, or acquisition of community oncology practices since 2008 [1,2]. This change has been driven by two major factors: the move from volume- to value-based payments, requiring more infrastructure management, resources, and market share as well as the rapidly increasing complexity of information needed to provide each cancer patient with the highest quality care while maintaining high patient and staff satisfaction, institutional health, and delivering these at optimal costs [3,4,5]. Meeting these goals has required innovations and teamwork that was pioneered first in several medical oncology home pilots from practices on the east coast by Dr. Sprandio and on the west coast by Dr. Bosserman as well as other practices across the US [6,7,8,9,10,11]. In the southwest, Dr. McAneny expanded on her New Mexico medical home model to engage six other US oncology practices in the successful Center for Medicare and Medicaid Innovation (CMMI)-funded COME HOME project [8]. Oncology pathways are a major component of these programs, with studies of implemented pathway programs showing equal or improved outcomes with lower costs in medical oncology [12,13,14,15,16,17], as well as in other disciplines including surgery, radiation oncology, supportive care, and end of life [18,19,20,21,22,23]. The evaluation and adoption of oncology pathways were further stimulated by Center for Medicare and Medicaid Services (CMS)’s oncology care MIPPs and Oncology Care Model (OCM) alternate payment programs and private payer pilots with academic and community-based networks and various private payors [24,25,26,27,28,29,30,31,32,33].

While multidisciplinary oncology pathway programs have evolved as essential components for evidence-based cancer care delivery within value-based cancer care (VBCC) programs, challenges remain to fully integrate and measure outcomes from multidisciplinary oncology pathways, including when they are combined into care plans across a patient’s cancer journey [34,35,36,37,38,39,40]. As a practical point, multidisciplinary pathways first must be adopted, implemented, and understood for each specialty before their combined impacts can be evaluated. The processes, teams, and tools to develop, implement, analyze, and iterate multidisciplinary pathway projects throughout a large enterprise remain a work in progress. We detail here the work to date by the City of Hope (COH) academic community network enterprise as an example of the methodology and tools, some well-established and some being pioneered, to implement multidisciplinary oncology pathways and outline many supporting projects that facilitate and optimize their impacts. We share available analytics which continue to evolve and expand in the hope that our work can help others in their transformation to VBCC.

## 2. Prioritizing Care Model Redesign for Value-Based Cancer Care (VBCC) at City of Hope with Multidisciplinary Oncology Pathways

**Figure 1:**
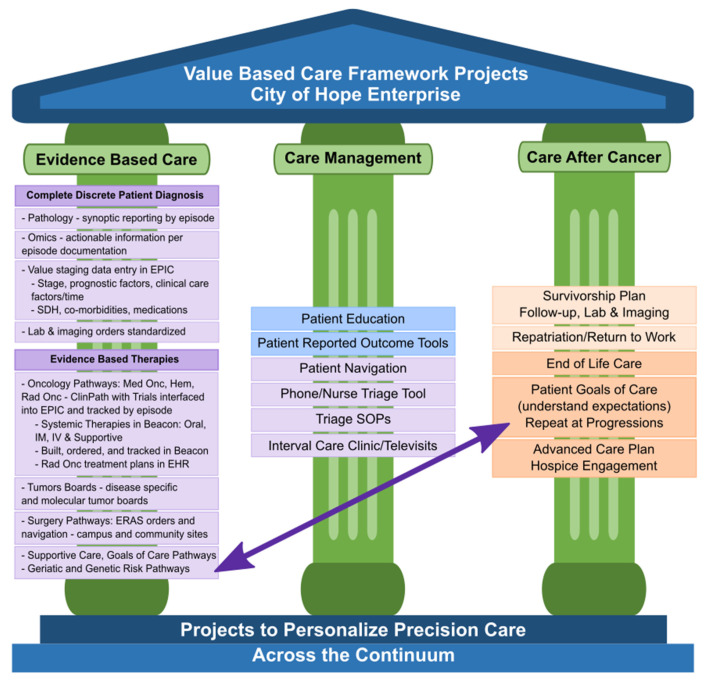
Value-based care framework projects for City of Hope Enterprise: Green represents the three pillars of value-based care: evidence-based care, care management, and care after cancer. The boxes represent categories of projects to facilitate measurable delivery of value-based care. Purple indicates clinician-led projects. Blue indicates patient-focused projects. Orange indicates projects after active therapy or curative therapy, whether in survivorship or end of life. Within the category of complete discrete patient diagnosis are the projects that will help to accomplish this. Omics refers to genomics, proteomics, metabolomics, and microbiome information that impacts a patient’s treatment episode choices and potential outcomes. SDH refers to social determinants of health. Med Onc—medical oncology, Hem—hematology, Rad Onc—radiation oncology. ClinPath is the pathway system by Elsevier (formerly called VIA Pathways). EPIC refers to the EHR. IM—intramuscular, IV—intravenous. EHR—electronic health record. ERAS—early recovery after surgery pathways. SOP—standard operating procedures. Televisits—telephone and televideo visits. The purple arrow indicates that goals of care pathways are incorporated during active therapy as well during in advanced end of life care.

In response to the City of Hope Enterprise leadership prioritizing the delivery of reportable value-based oncology care, a Value Realization Project (VRP), led by a multidisciplinary group of clinicians, pharmacists, nurses, administrators, informaticists, program managers, quality, data analysts, risk and outcome experts, and our chief medical and administrative officers, was formed. This VRP group developed a Value Framework (VF) with specific projects identified under each of the three pillars of evidence-based care, care management, and care after cancer, as shown in Figure 1. These pillars are derived from the key components identified in the medical oncology home and OCM models as well as several value-based payer initiatives which have shown value [6,7,8,11,23,24,25,26,27,28,29,30,31,32,33]. A full discussion of our value-based care initiative is beyond the scope of this paper; however, we present our value framework and highlights of several other key projects within the framework to show the interdependence of those projects in advancing the implementation and evolving outcome analytics of multidisciplinary oncology pathways. Interdependent projects include the capture of complete staging data in the electronic health record (EHR), systemic therapy regimen orders built into the EHR, integration of clinical trials and pathway decision support into the EHR, and incorporation of precision medicine, among others. These companion projects support measurable evidenced-based cancer care delivery. We discuss some of the pathways and projects in the other two pillars of VBCC: management of care while on active treatments, and care after active cancer, whether in survivorship or at end of life, as part of our COH supportive care programs. Not shown in Figure 1 are the enterprise-wide initiatives to engage and measure patient and staff satisfaction, institutional health as well as the administrative support needed in informatics, precision medicine, EHR enhancements, care models, and payer contracting to fully meet VBCC goals. As we build each of the identified projects supporting multidisciplinary oncology pathways, we are working on measuring individual pathway use and impacts while working toward measurement of the larger clinical, financial, clinical trial, and quality outcomes. We also continue to iterate methodologies for team-based care and patient engagement trough academic and community staff teams in the evolution toward a mature VBCC model.

## 3. NCCN Guidelines and Growing Complexity of Evidence-Based Cancer Care Also Stimulated Multidisciplinary Oncology Pathway Development at COH

City of Hope (COH) is a National Cancer Institute (NCI)-designated comprehensive care center and one of the original 13 member institutions that formed the National Comprehensive Cancer Center Network (NCCN) in January of 1995. Within the NCCN, our faculty have participated in and led the development of guidelines for the treatment of cancers for the past 25 years. The COH enterprise now serves patients with cancer and metabolic diagnoses in a region of 25 million residents living in five counties in Southern California as well as more distantly based national and international patients. The COH enterprise includes a central academic, clinical research, hospital and clinical care campus in Duarte, California and a clinically integrated, community practice network of 31 community practices. Approximately 30% of COH physicians practice in the community (either solely or in a mixed hybrid of community/academic practice). Clinicians in the community are mostly general oncology practitioners, while the academic clinicians are disease and disease subtype specialists. Over the past year, we have identified community doctors who have a special interest in a specific cancer and will see patients with that disease preferentially (referred to as disease-leaning), such as breast, thoracic, gastrointestinal, and genitourinary cancers. A single foundation faculty model employs both academic center and community clinicians. The mission of the organization is to develop and bring cutting-edge precision cancer care and clinical research to every patient that is also personalized and delivered with compassion, high quality, and cost consciousness within the value-based cancer care initiative.

Although COH faculty participated in the development of the original eight disease guidelines published by the NCCN in November of 1996 and continue to participate and lead disease guideline committees at NCCN, until the more recent partnership between NCCN and US Oncology and their development of Level 1 Pathways powered by NCCN, the NCCN guidelines were broad and without the detailed pathway approach to better guide individual patient care [32]. COH disease teams regularly discussed narrowing the NCCN guidelines into pathways for specific patient issues and cancer subtypes and some, such as the breast, lung, and renal teams, develop and review pathways that are shared with COH faculty, as described in companion articles in this series [41,42,43]. With the growth of the COH enterprise, however, the need to more formally establish not only pathways for each disease but the ability to prompt for those choices, keep the pathways up to date, incorporate clinical trials as well as track the use and variations of treatments for each patient (by disease, disease subtype, clinicians, site, and payer), a formal pathway program became a priority. As part of the VBCC initiative, available pathway programs and options were evaluated by a multi-stakeholder group of faculty, staff, nurses, and administrators with campus and community network representatives over a 2-year period. This was led early on by an oncology surgeon who practiced at both the academic center and in the community and understood the practical needs for a tool to guide standardized care for similar patients. Work initially focused on the development of multimodality clinical care pathways, defined as standardized, evidence-based interdisciplinary care management plans, which would identify an appropriate sequence of clinical interventions, timeframes, milestones, and expected outcomes for a comparable patient group, i.e., by diagnosis or surgical procedure. As the work evolved, however, it became clear that the starting point for these larger multimodality care plans should be pathway programs starting with the specialties of medical, radiation, and surgical oncology.

A formal pathway tool became a priority not only to support the measurable delivery of care across the growing COH enterprise but to bring expert input to the point of clinical care for the growing number and complexity of cancer therapies. The continued growth in new Food and Drug Administration (FDA) hematology–oncology drug approvals since 2000 is a telling example of the need to provide real-time expert decision support across an enterprise to aid the delivery of high-quality, high-value cancer care. The FDA website for oncology–hematology drugs reports that from 2016 through June of 2020, 230 new drug approvals were issued, up from 22 in 2016 to 58 in 2017, 63 in 2018, 46 in 2019, and 41 in the first half of 2020 [44]. These approvals are also increasingly complex and include not only new drugs but new indications for existing drugs. The approval can be under the expedited review process so that the approval could also change with further data. Approvals can include whether the use is approved in early disease, as neoadjuvant, adjuvant therapy, or extended adjuvant or for advanced disease. The approvals can include use only with specific biomarker results from the patient or their tumor, and sometimes only after biomarker testing by specific laboratories. The approvals often specify use only in specific lines of therapy, after previous general or specific therapies, in combination with specific other drugs or drug categories. Biosimilar drugs are also listed in the FDA approvals with their specific uses referenced to the reference drug [44]. Thus, keeping up with the details of the almost weekly new FDA approvals, which individually can impact one or many kinds of cancers, requires increasingly sophisticated and constantly upgraded decision support pathways not only for clinicians but also for patient education to support their participation in shared decision-making and to ensure appropriate use and documentation requirements for authorizations and payer coverage. Financial toxicity has been identified as another growing barrier to patients receiving appropriate cancer therapies, especially targeted, molecular, and immunotherapy-based treatments [45]. Thus, building the workflow infrastructure to support the use of pathway tools which include increasingly higher cost therapies can also support more timely authorization of treatments for patients and can help to engage early patient assistance, when needed, so patients can receive timely care which improves outcomes [46,47]. Several examples of the growing complexity of high-quality care are described in companion articles on colorectal and non-small cell lung cancer by colleagues in this series [48,49].

The development of surgical pathways was driven by the benefits of standardizing surgical type and processes but also by the benefits of reducing morbidity, mortality, and costs by coordinating education and care processes with the many providers involved in a patient’s care before, during, and after surgery [50,51,52,53]. These comprehensive care plans have come to be known as Early Recovery after Surgery (ERAS) pathways [54,55,56,57]. At City of Hope, ERAS pathway work began in the early 2000s by the urology faculty with the development and implementation of a successful ERAS pathway for cystectomies showing lower length of stay, complications, and readmission rates that were confirmed by outside groups as well [19,58,59]. This stimulated the development of further urology ERAS pathways as well as ongoing work on ERAS pathways for other surgical subspecialty departments for the COH academic campus and at regional hospitals where COH faculty practice.

Given these many drivers toward standardizing care in all specialties that could provide better care, better outcomes, and potentially lower costs, City of Hope’s Value Realization Project identified a Value-Based Core (VBC) team of experts to meet regularly and work with a multidisciplinary physician VBC group team, using the Value Framework projects noted in Figure 1 above, to engage disease leaders in medical oncology, surgery, and radiation oncology along with administrators, information technology (IT) and informatics and EHR experts to evaluate, implement, and iterate oncology pathways for the enterprise. Insights and outcomes from this work follow and add to the work of other academic community network enterprises working together to improve the quality of care for cancer patients [60,61].

## 4. Medical Oncology, Radiation Oncology, and Hematology Subspecialty Pathways

### 4.1. Evaluation and Adoption Process for Pathway Processes and Tool for the Enterprise

**Figure 2:**
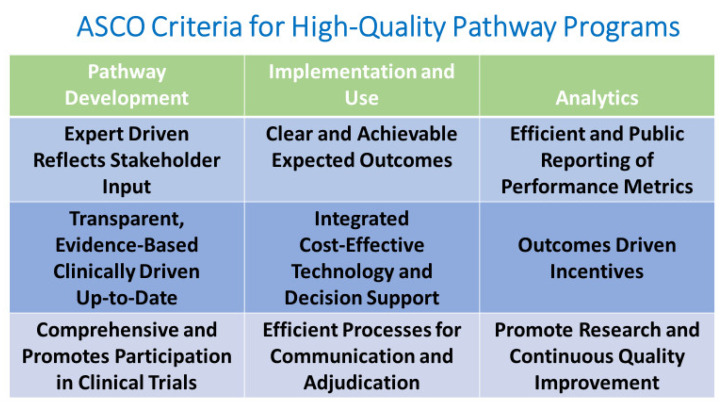
ASCO’s criteria for high-quality oncology pathway programs under pathway development, implementation and use, and analytics as described by Zon et al., J Oncol Prac 13:207–210, 2017 [63].

Initial meetings identified medical oncology pathways as the starting point for a formal oncology pathway tool. Available oncology pathway programs, their implementation, and integration abilities were evaluated during regular multi-stakeholder meetings. In 2016, a decision was made to use the VIA Oncology Pathways developed at the University of Pittsburg Medical Center (UPMC) Hillman Cancer Center (now ClinPath by Elsevier and will be referred to as such going forward) which met the criteria of the January 2015 ASCO Policy Statement on Clinical Pathways in Oncology as well as their criteria for high-quality pathway programs shown in Figure 2 [62,63]. The ClinPath pathways were then evaluated and approved by each medical oncology disease team for a 1 January 2017 go live date. The ClinPath system was chosen because it addressed the most common tumor types with readily available evidence summaries for clinician review and prioritized pathways by efficacy, toxicity, and cost [64]. The pathways were being recognized by payers as part of growing value-based payer initiatives [16,17,30,31,32]. The ClinPath Pathway program also welcomed our faculty to actively participate and, when appropriate, lead pathway disease committees. The regular disease committee meetings, made up of national pathway disease experts and interested users, work to keep pathways updated regularly. In addition, pathways were available for hematology and radiation oncology, with plans for further expansion of pathways for additional solid tumors, hematology, radiation, and possibly surgical pathways. A computer icon tool could be launched from campus and community computers on different EHR platforms for the January 2017 go live with the capability to be later integrated into the planned enterprise wide EHR change to the EPIC system (EPIC systems, Verona WI) in December of 2017.

### 4.2. Initial Medical Oncology ClinPath Adoption Processes and Definitions

Following the decision in 2016 to implement ClinPath pathways, starting with medical oncology, teaching decks were built by the COH pathway and education teams to share the rationale, clarify the diseases for initial navigation at go live 1 January 2017, and share use and development expectations. On-pathway vs. off-pathway treatment decisions were defined. On-pathway decisions were decisions that adhered to the pathway’s decision algorithm for that specific stage of disease. Clinical trials and secondary treatments for alternate patient scenarios were still considered to be on-pathway, as were any decisions to not treat or to take a patient off active treatment. Off-pathway decisions that did not align with pathway recommendations on review were typically driven by new data not yet incorporated into the pathway (indicated as physician disagrees with pathway choice or a free texted comment to that effect, reports not shown), unique patient presentations, specific patient preferences, and on occasion by physician discretion. It was emphasized that while it was expected that for most cancers, on-pathway choices would be in the range of 80% or more, there would never be an expectation of a specific pathway compliance percentage by doctor or by disease type. It was also recognized that in these times of rapid new drug discovery and molecular targeting, the best therapy for an individual patient may not yet be in the pathway tool. All clinicians were encouraged to join one or more pathway disease committees to participate in the ongoing development process, review the latest evidence, and share best practices.

### 4.3. Key Milestones and Practical Time Implementations for ClinPath Pathways across the COH Enterprise

January 2017: Medical Oncology go live for six disease types: Medical oncologists at the academic campus and community sites were trained and instructed to use the pathway tool over 6–8 weeks prior to go live for all new medical oncology therapies including both initial therapy and subsequent therapies in six disease categories: breast, lung, genitourinary (GU), gastrointestinal (GI), gynecologic (GYN), and head and neck. Educational tip sheets and training team personnel were available on an ongoing basis for individual support as well. Other solid tumor types and some hematology diseases were available in the tool but optional for use and not assessed as part of our initial metrics. COH had two EHR systems when the VIA pathways went live: Allscripts on campus and Touch Works in the community. Both were interfaced to pull patient demographic and physician schedule information into the pathway tool. After that, however, clinicians would navigate pathways by entering discrete staging and prognostic or clinical care feature elements to reach the recommended pathway choice or choices. Physicians were required to enter a reason when an off-pathway decision was chosen. After making a therapy choice, clinicians then went back to their EHR to order the chosen therapy. Clinicians were made aware of plans for future EPIC integration, addition of clinical trials into the pathways, and expansion of tracking and reporting for other disease types and specialties. Although formal surveys were not done, clinicians expressed frustrations from the double entry of data from their EHR notes into the pathway system, having to use the system when they were confident and familiar with the appropriate pathway choice as well as skepticism on future upgrades and benefits to their disease programs (personal communications).

2 December 2017: Enterprise-wide transition to Epic: Treatment protocols were built in Beacon by the disease teams based on the regimens used in the previous systems and a review of those in the pathway system before go live. Additional COH-specific regimen preferences and many clinical trials were also built. Oral chemotherapy regimens were not prioritized. Standardized nausea regimens based on NCCN emetogenic levels were added for each regimen as well as hypersensitivity premedication regimens and they were integrated when needed to avoid overuse of steroid medications. All documentation, data entry, and ordering were then done in the EPIC EHR across the enterprise.

January 2018: OnCore clinical trial management system went live. Its benefit to the pathway program is discussed below.

August 2018 and ongoing: Clinical trials were formally added into the ClinPath pathway tool by disease teams starting with the most commonly seen cancers in the community. Additional solid tumor clinical trials have been added and closed trials removed as noted below.

March 2019: Radiation oncology clinicians on campus and community sites went live on ClinPath radiation pathways with integration of COH radiation oncology clinical trials as described below.

20 July 2019: The one-way integration between the Epic EHR and the pathway tool was started and is described below.

August 2020: Hematology pathways had been available in the ClinPath system but their formal evaluation by disease leads began as well as identification of clinical trials to be added to the ClinPath pathways. As reviews are being completed, trials are being added and formal launch of enterprise-wide use and measurement of ClinPath for the common hematology diagnoses is scheduled to start December 2020 as described below.

6 October 2020: The two-way integration between the EPIC EHR and the ClinPath tool went live in the EHR for 11 disease subtypes (breast, bladder, colorectal, gastroesophageal, melanoma, mesothelioma, ovary, prostate, testicular, small cell lung, and thyroid). With two-way integration, we expect that most if not all discrete staging elements from the EPIC staging forms will be interfaced to auto-populate into the ClinPath tool over time and as elements are added to the EPIC system. Any additional or missing discrete data elements in the EHR system would still have to be entered into the pathway tool to trigger a pathway prompt. At go live, tumor types fell into three categories from this pioneering work: fully mapped tumors (breast, colorectal, gastroesophageal, melanoma, mesothelioma, bladder, ovary, sarcoma, small-cell lung cancer, testicular, thyroid, and uterus), partially mapped tumors (head and neck, prostate), and pending mapped tumors (anal, non-small cell lung, neuroendocrine, pancreatic, renal). When clinicians navigate within the EHR to the ClinPath system, the data elements entered into the EPIC staging forms are then shown on a field next to the ClinPath data entry choices. For fully mapped tumors, one click of an APPLY button automates the entry of the EPIC data into the ClinPath system. The doctor then continues navigating to the pathway choices. For a partially mapped tumor, those elements that are mapped will auto-populate in ClinPath and the others will have to be manually entered. For non-mapped tumors, the EPIC data are there to see but each element has to be manually clicked before continuing the navigation. Whether or not a tumor is fully, partially, or not yet mapped, however, after a pathway choice is made, whether for a clinical trial or a standard regimen, the user is then taken back to the EPIC Beacon regimen for completing the order or ordering a clinical trial team evaluation. This two-way integration will continue to expand by matching additional data elements captured in EPIC to the pathway decision trees to more efficiently guide pathway choices. In addition, the EHR system is working to include data elements that more fully describe a patient’s disease status as required by decision support tools. It is expected that ongoing work will continuously reduce duplicate data entry requirements, including for the growing number of actionable genomic results, while ensuring that the primary clinical information remains in the enterprise EHR [65]. A survey of provider experience, time savings (if any), and satisfaction was built and, pending team approval, will be sent to all faculty who use the ClinPath system in November of 2020 to further inform this work.

### 4.4. Clinical Trials Incorporated into ClinPath System and Integrated with Clinical Trials Management System Can Prompt for Available Trials and Track Adoption

Studies have shown improved clinical trial assessment and accruals for clinical trials incorporated into pathway tools that are routinely used, including by multisite organizations [66,67]. The ability to add clinical trials available at and through our COH enterprise was another key reason the ClinPath pathway system was chosen in 2016 [68]. Prior to incorporating clinical trials into our pathway tool, though, COH implemented the OnCore clinical trials management system (CTMS) in January 2018. This system provides faculty and clinical research teams with a comprehensive integrated system that supports virtually all aspects of clinical trial offerings including features for managing studies, electronically capturing protocol and patient data, creating custom reports, and supporting financial activities. Having it fully linked into the decision support pathway tool enhances clinician notification of available clinical trial options, especially timely notices of pending, open, on hold, or closed trials to maximize clinical efficiencies.

The OnCore tool integrated with the EHR will hopefully enhance the value of the pathways tool through bi-directional data flows indicating protocol status and patient demographics. Phase I of the project included the decommissioning of the legacy clinical trials management system (MIDAS) and migration of protocol status and data. Phase II of the project linked detailed subject management activities enabling COH to track patient visits, enhance charge segregation, and payment reconciliation. OnCore was also integrated with City of Hope’s regulatory committee management platform, iMEDRIS to provide greater efficiency and data integrity. Phase III, which is going live Q3 of 2020, involves migrating and making available to investigators the IRB-approved versions of the protocol-related documents and informed consent forms in OnCore and decommissioning our current Clinical Trials On Line (CTOL) system, where these documents are currently. This will reduce the number of systems which investigators will need to navigate.

A major goal of an integrated decision support pathway tool is the incorporation of clinical trials prompted by the patient’s disease information. After the initial pathway tool launched in January of 2017, training was reinforced, navigations increased but both campus and community faculty were eager to have clinical trials placed into the pathways. The pathway team PharmD (DJ) collected a list of clinical trials from each disease lead, worked with them to place each trial in the appropriate pathway branch points, then submitted the information to the pathway company for inclusion in the tool for all faculty at all COH sites. This process took 4–5 weeks for each disease. The schedule and disease types for availability of the clinical trials in the pathway tool went as follows: GI: 8/2018, 15 trials; Lung: 2/2019, 15 trials; GYN: 5/2019, 5 trials; Breast: 7/2019, 18 trials; GU: 9/2019, 20 trials’; Head and Neck: 4/2020, 12 trials.

Still pending disease types for adding clinical trials in solid tumors are melanoma, sarcoma, and neurologic tumors. With the hematology division now actively engaged in finalizing their adoption of the ClinPath hematology disease pathways, disease leads are working with the pathway PharmD lead to have their clinical trials added into the pathway tool for the planned launch of enterprise-wide use of the hematology pathways in December of 2020. Doctors, APPs, and disease leads have informally but almost universally expressed anticipated value in improving clinical trial identification for their patients as they navigate their therapies in the pathway tool.

**Figure 3:**
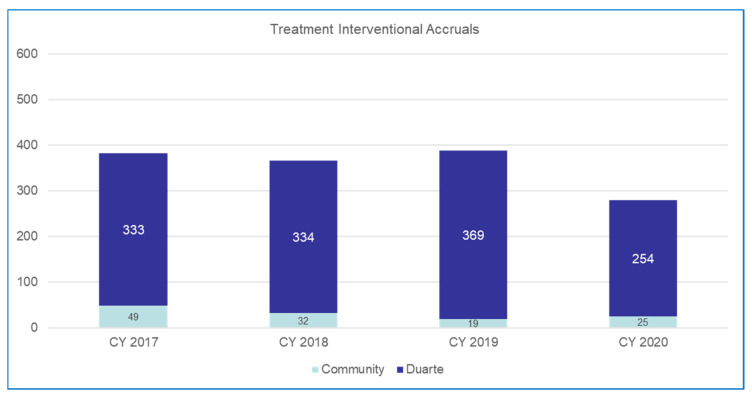
Clinical trial accrual at Duarte and community sites 2017 through September 2020. Light blue bars are the number of patients accrued to treatment trials at community sites where some trials were available. Dark blue bars are number of patients accrued to treatment trial at the Duarte academic campus.

Pathway reports show 4.5% of patients by individual medical record number were on a past or present clinical trial from the start of our pathway use in January of 2017 until June of 2020 for the six originally monitored cancers (GI, Lung, Breast, GU, GYN, and Head and Neck). The per quarter percent of patients on a trial varied from 2% to a high of 7–8% in Q4 of 2018 and Q1 of 2019 after the GI and lung cancer trials were added to the pathways. The impact of the full integration of clinical trials into the pathway tool will not be fully assessable until additional programs are matured during 2021–2022. These include the ongoing expansion of clinical trial hubs into regional community sites, which started in 2020 and will cover most of the network regions in the next 2 years. Figure 3 shows that our overall accrual numbers remained similar overall in the community sites and the academic center between 2017 and 2019. The academic accruals include community patients referred to the center who entered a clinical trial. The overall numbers for 2020 reflect only 9 months of data which are promising, given the reported major decreases in clinical trial accruals across the US since the COVID-19 pandemic began in March 2020. As the community network clinical trials programs expand with regional trial directors and trial hubs, plans to open more trials for eligible patients in the community, and the addition of hematology trials for the common hematology diseases frequently seen in the community sites, we expect that the inclusion of clinical trials in the ClinPath pathways will stimulate increased assessments and accruals for community network patients over the next 2 years. The expansion of the discrete data capture in the EHR system and pathway navigation reports will also be fed back regularly to disease teams and the clinical trial leadership so that they can better track the impact of assessments and accruals for clinical trials across the enterprise. The ability to track every patient who goes on a systemic therapy in hematology and medical oncology as well as a radiation oncology treatment plan will allow trial leads to more quickly identify gaps in referrals that can then be addressed. Additional issues are reviewed by colleagues in an accompanying article in this series [69].

### 4.5. Oversight and Insights from ClinPath System Pathways Use in Medical, Hematology, and Radiation Oncology at COH

The ClinPath pathways program at COH was and is overseen by an interdisciplinary team which continues to meet two or more times per month. This team reviews and directs data analytics, gathers feedback from clinicians and administrators, directs dashboard and interface development, sharing of reports to individual clinicians, site and disease leads as well as administrative and contracting leadership. They oversee and encourage ClinPath disease committee and leadership participation as well as ongoing clinical trial and EHR integration with expansion of pathway adoption across diseases and disciplines. The group reviews monthly pathway compliance rates to identify outliers, leading to further study of clinical issues, patient issues, or individual faculty issues that alter compliance rates, most often because new data have not yet been incorporated or there is not a pathway for an individual’s episode of care need. COH faculty’s participation and, for some, leadership of disease committees has been essential in supporting timely updates of practice changing information as well as sharing back information and the national perspectives on care standards.

Utilization of the pathways by COH clinicians has increased over time and serves as one of the performance metrics for members in medical oncology. These metrics are reviewed by the leadership (including the chairs, senior medical director of community practices, and regional medical directors of community practices) as well as the COH pathway and the value-based leadership teams to maximize standardization, quality, and value of clinical care across the enterprise.

Clinicians, especially those in the community, report that the availability of the pathways has improved clinician efficiency and time management. With the knowledge that these are standardized pathways, agreed upon by the entire institution, with the added benefit of integrated clinical trials, there is less need to contact disease team academic leader(s) for many opinions, even in these times of increasing availability of newer therapies and clinical trials.

While doctors are navigating new therapy starts consistently in the pathway tool, the COH pathway team was asked to track the correlation between indicating a pathway choice in the tool and ordering the regimen in the EHR as a quality control measure. A Variation of Care report was designed in 2019 and reports the monthly rates of any difference between what was navigated in the ClinPath system and what was ordered in the EPIC EHR through the Beacon module. The data from May to December of 2019 showed that the difference varied from a low of 0 variations from 871 navigations in 11/2019 to a maximum of 18 variations from 688 (2.6%) navigations in October of 2019. Overall, discrepancies were rarely over 3%. A random chart evaluation noted that these discrepancies occurred when patients or providers changed their treatment plan based on additional work-up, most often when additional disease was found. They then ordered the therapy in the EPIC EHR Beacon treatment orders but did not go back into the ClinPath tool to change the navigation information in the pathway tool. This also happened for rare patient preference for an alternate therapy after the initial ClinPath navigation was done.

**Figure 4:**
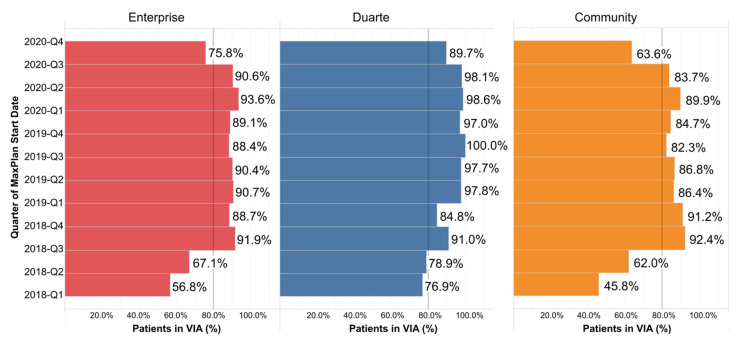
Percentage of new EPIC Beacon starts that were navigated in ClinPath by medical oncologists for solid tumors since go live for enterprise overall then by Duarte campus site and all community sites. Note Q3 data were incomplete at time of manuscript data collection in May 2020, so the final% of patients in Q3 who were navigated in ClinPath is likely higher, as is seen in other recent quarters.

Since our ClinPath go live in January of 2017 until early Q3 2020 when data were reported for this manuscript, clinicians had done 28,271 total navigations, including 16,034 navigations from the academic clinicians and 12,190 navigations from the community clinicians, including 47 at our newest Newport Beach site, opened in January of 2020. The percentage of therapies ordered in Beacon that were navigated in the pathway tool by enterprise, community, and campus doctors since implementation is shown in Figure 4. Pathway navigation was higher on the academic campus except for two quarters. The reasons are under further evaluation.

Simply having a pathway system does not ensure the use, usefulness, or production of informative analytics. A multidisciplinary pathway team of clinicians, analytic, quality, her, and informatics experts meets every 2 weeks to evaluate pathway use, compliance by disease type and clinician, track low compliance issues to understand if a new therapy not yet incorporated into the pathway or other reason is increasing off-pathway choices, use of clinical trials, and whether the indicated therapy in the pathway system is, in fact, the ordered therapy in the EHR. An enterprise-wide incentive program to capture pathway use and choices for medical oncology was launched in 2020 and final percent navigations in the pathway for all medical oncology therapies in the EHR trends have risen significantly. Disease leads have also requested regular reporting of every patient who meets specific disease, stage, and other criteria for an available trial as to whether or not the trial was considered and if a trial evaluation and ultimate accrual occurred. Several leads are asking for real-time reports so that they can more proactively reach out to network clinicians to resolve any barriers for patient accrual to an available clinical trial. Ultimately, by tracking specific clinical trial eligibility and accrual by site, disease, and trial type across the enterprise, the trial team can optimize which types of trials are opened at which sites to maximize clinical trial resources.

**Figure 5:**
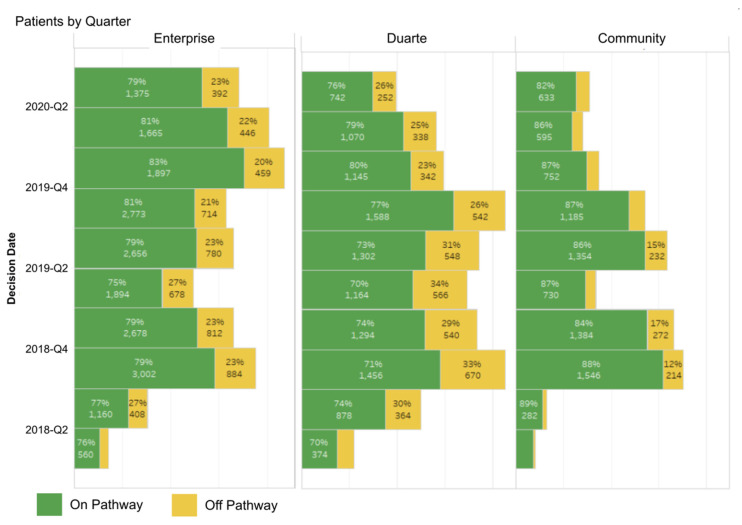
Pathway compliance percentage over time for medical oncology patients navigated in pathway tool (ClinPath) for enterprise, Duarte academic campus, and community sites for new therapy regimens ordered in the EPIC-BEACON EHR from January 2019 through June of 2020. Green bars are on-pathway choices (including clinical trials) and yellow bars are off-pathway choices. Note that the scale of the x-axis representing the numbers of patients is different for each group to show the comparison percentages in one graph. The combined number of patients navigated in Duarte and community sites is reflected in the number totals described on the enterprise graph.

Having a pathway tool enables the tracking of the percentage of patients who are treated on-pathway (including clinical trials) and off-pathway across the enterprise and by academic vs. community network sites as shown in Figure 5. Academic clinicians consistently see more patients treated off-pathway, which, on reviews, has been explained by the higher number of patients seen where a pathway does not exist for the patient presentation or where a different treatment is recommended due to new molecular findings or emerging data are not yet incorporated into the pathway. After reviewing data, the pathway committee has identified and recommended to the pathway vendor that they add that additional reason as an option in the dropdown list when entering a reason for choosing an off-pathway therapy. Such a new choice for going off-pathway, “new information not yet incorporated into the pathway” could then be tracked for each tumor type and stage during six month intervals over twelve to twenty four month periods to see if and when such a therapy choice is added to the pathway. This could also better inform clinicians about how rapidly new, practice-changing therapies that experts agree should be used become available for prompting in the ClinPath decision support pathway tool.

### 4.6. Pathways for Radiation Oncology Using ClinPath Program

As with medical oncology pathways, radiation therapy pathways were piloted by the University of Pittsburgh Medical Center (UPMC) group, who led the development of the ClinPath (then called VIA) pathways for radiation oncology based on the same principles used for the development of medical oncology pathways to standardize care for the most common radiation treatments, prioritizing efficacy then toxicity then costs. Studies by their group showed that using the pathways with a peer review process appeared to encourage compliance with clinical pathway recommendations [70]. Specific studies included one showing increased community practice physicians’ adoption of hypofractionated regimens for whole breast radiation [71].

This is significant as several studies have shown slow and or poor adoption of evidence-based use of shorter course radiation treatments which have lower costs and shorter treatment times for patients with equivalent outcomes. Hypofractionation for breast cancer is a good example. While the majority of women undergoing radiation therapy for early breast cancer have equal disease control with equal or better cosmetics and a shorter course for patients has been adopted by UK and Canadian clinicians for the majority of patients, it has been much slower to be adopted in the US despite positive long-term outcomes in studies and expanded guidelines for use by the American Society for Radiation Therapy (ASTRO) [72,73,74,75]. As in medical oncology and hematology, the growing options for the use of different radiation techniques such as 3D conformal, intensity-modulated radiation therapy (IMRT), proton beam, and other methods of localized radiation therapies along with shorter treatment courses have led to increasing use of radiation therapy pathways to support busy clinicians through their prioritizing efficacy, toxicity, and safety as another component of value-based care [76,77].

As the COH enterprise expanded with many satellite sites delivering convenient local radiation therapy treatments, the radiation oncology department chose to implement and use ClinPath radiation pathways at all sites in March 2019. COH has the largest single institution radiation oncology network in California, comprised of 46 physicians practicing at 20 satellite sites. ClinPath pathways were integrated into the day-to-day clinical practice for 35 radiation oncologists at 16 sites as four outside hospital site doctors are not yet on the system. Radiation oncologists with specific disease interests contributed to the development of some of the pathways and some faculty now co-lead some radiation oncology disease pathway committees. Clinical trial options were also integrated into the pathway options customized to our institution.

The radiation oncologists use the EPIC EHR for all care, except the specific radiation therapy planning, which is done in the radiation-specific modules of MOSAIQ or ARIA EHRs at different sites. The pathways have had a launch button integrated in the EPIC EHR and the radiation oncologists are looking forward to the two-way integration in progress so that the staging in EPIC will be pulled into the pathway system which is on the planning agenda for 2021–2022.

The pathways, besides giving case-by-case feedback regarding treatment options to clinicians, also give detailed recommendations regarding the technical aspects of radiation (i.e., dose constraints, fractionation options), which is useful at the time of actual radiation treatment planning. The pathways cover the most common disease sites and scenarios. There are still complex or rare cases that do not fit into any of the existing pathways, where the radiation oncologist notes “no pathway”, and there is an option to add a narrative comment.

**Figure 6:**
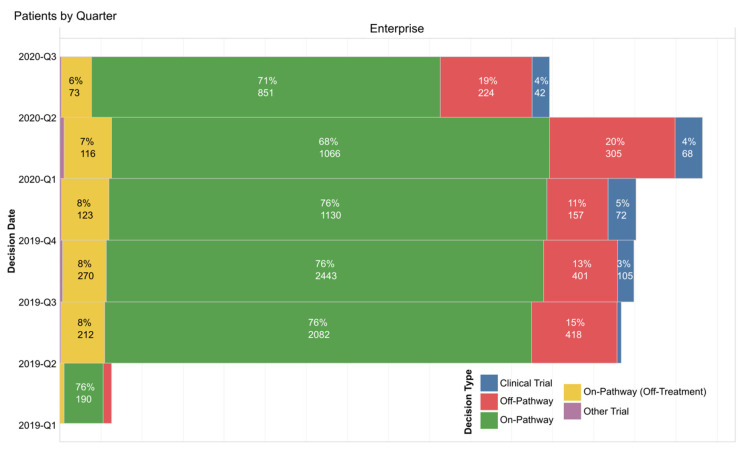
Individual patients seen by radiation oncologists by the enterprise per quarter since ClinPath pathways were initiated for radiation therapy in Q1 of 2019. The numbers represent the number of individuals with a decision made each quarter who are: on therapy with a non-pathway diagnosis, shown as “other trial” (purple), were on a pathway but went off treatment (yellow) that quarter, on a pathway treatment (green), are off treatment (light blue), are on an off-pathway treatment, (red) or on a clinical trial (dark blue) for an on-pathway disease.

In general, the pathways program has been well received. There has been >90% participation rate by COH radiation oncologists in using the pathway tool to capture their treatment decisions, with most physicians using it to indicate 100% of their decisions. Physicians are also sent auto-generated weekly reminders listing any patients where pathways data about their treatment choice were not entered. COH does not require strict adherence to the pathway’s preferred choice, but the analytic team gives physicians feedback which can show clinicians where their choices fall and how they compare to their peers. On- and off-pathway rates are tracked for feedback to individual clinicians, disease, and regional site leads. Pathway compliance remains high after initial launch and has been at or above the generally accepted 80% pathway compliance benchmark, which is an accepted standard in the radiation oncology quality assurance program as defined by the quality assurance review center (QARC), which established an 80% rate based on clinical trial standards [78]. The COH on-pathway rates for the combined enterprise and the separate Duarte and community sites are available but we share in Figure 6 how available additional reporting can further detail the types of therapy or trial as well as who has just completed a therapy plan or who is off therapy by quarter for all patients seen. This same graphic can be constructed separately for Duarte campus radiation therapy, for all as well as for individual sites and by doctor.

Individual radiation oncologists informally report at leadership meetings that they have found the pathway information regarding various fractionation options helpful. Feedback and questions regarding pathways content are welcomed at all levels but formal feedback from radiation oncologists has not been solicited. COH faculty working at the main medical center and satellites engage via many regularly scheduled virtual meetings both before and exclusively after the COVID-19 pandemic from March 2020 forward. They have weekly peer review of patient new starts, monthly didactic sessions regarding a specific disease site where a COH attending reviews a case and asks residents questions, anyone can contribute, and is extremely well-attended. They also have weekly breast and head and neck tumor boards and include community physicians on disease teams. The entire radiation oncology department has previously gotten together for a yearly in-person one-day science and clinical care retreat, which will be held virtually during the pandemic. Although no single physician has time to participate in every discussion, the regular meetings are available with technology support. Clinical leaders note that it remains challenging to engage and include new and distant satellites as they continue to be added to the COH network, but this is an identified priority.

The COH radiation oncology department has also created an internal Microsoft Teams messaging system where anyone can share case questions and message the entire department. It has yet to be regularly utilized but doctors are in the process of being trained on the Microsoft Teams functionalities. E-mail, therefore, is still the most utilized form of communication department-wide. Overall, pathway compliance is high for the enterprise, as shown in Figure 6 above. Reasons for off-pathway choices are captured as for other specialties, reported and reviewed by the disease teams. Any recommendations for pathway updates are communicated to the appropriate ClinPath disease committees.

### 4.7. Additional Multidisciplinary Pathways for VBCC: Geriatric Oncology and Genetic Counseling/Risk Assessment

Geriatric oncology is an established field pioneered by the late COH faculty member, Dr. Arti Hurria. Dr. Hurria’s goal was to bring the principles of geriatric oncology into practice through standards and pathways to improve the care of older adults with cancer. Given that one in three Americans aged 60 years or older will be diagnosed with cancer in their lifetime and the population of older adults is growing rapidly with the aging of the U.S. and worldwide populations, cancer incidence is expected to rise by 67% for individuals aged 65 or older between 2010 and 2030. Although older adults represent the majority of individuals with cancer, they are severely under-represented in clinical trials and research. Hence, the majority of evidence that sets the standards of care for oncology treatment pathways is derived from younger individuals.

To address these unmet needs, COH formed the Cancer and Aging program in 2006, under the leadership of Dr. Hurria, which ultimately led to the formation of the Center in Cancer and Aging (CCA) in 2017. The mission of the CCA is to join investigators from all cancer disciplines to study the biology, treatment, and survivorship issues of older adults with cancer. Over the last decade, COH staff have led over 30 studies in cancer and aging, enrolled more than 5000 patients on studies, disseminated research findings through more than 250 publications, and received competitive grants from the NIH as well as philanthropic funding. CCA is one of the elite centers in cancer and aging in the nation.

With this background work, COH’s CCA is now leading the way in expanding pilot pathway projects into community sites, starting with standardizing geriatric assessments (GA), which can help to guide personalized care for our increasingly aging population while achieving their best health outcome. For example, an ongoing implementation study of an APP-led multidisciplinary team telehealth intervention is being conducted in one of COH’s 31 community cancer sites. This Antelope Valley regional site is in a remote area of high need and limited resources. Learnings from this pilot will be used to expand geriatric pathways for assessments and care planning across our enterprise.

Colleagues at COH have also examined the multifaceted barriers hindering participation of older adults in cancer trials. They offer strategies to improve the participation of older adults in clinical trials in an accompanying article in this series [79]. It is expected that many of the projects outlined in COH’s Value Framework, such as the complete discrete staging and collection of social determinants of health along with geriatric assessments with clinical trial integrations into the pathway navigation tool for systemic therapies in medical oncology, hematology, and radiation oncology, will help to support the piloting of these strategies.

Clinical cancer genomics has been an expertise at COH since the department was established in 1996 which has grown to provide training, research, and consultative services overseen now by the division of clinical cancer genomics (CCG). A counseling, research, and professional teaching program, internationally recognized for its onsite and telehealth training of healthcare providers worldwide, continues with ongoing professional conferences open to trainees and the COH faculty at all sites. At the academic center, the department provides consultations for individual cancer risk assessments, genetic testing and result interpretation, risk reduction counseling, and clinical trial enrollment. The CCG division is also engaged in the support of COH’s enterprise precision medicine initiative to expand somatic and germline testing with associated counseling on results, integration of results into care pathways and into research teams to power new discoveries. Work to standardize these processes through discrete data entry in EPIC on the upgraded staging forms is in progress, along with ways to present clinically actionable results of expanding somatic and germ line genomic testing to clinicians within the data for each patient’s episode of cancer throughout their journey. Having relevant clinically actionable data for integration into decision support pathway tools and molecular tumor boards while having the larger dataset easily accessible for analytics and discovery by the research teams is a current priority of the precision medicine, genomics, informatics, value-based care, and disease specialty teams informed by input from faculty at both the academic and community sites.

Genomics pathway work is in the earlier stages of development and will be informed by the expansion of CCG services to the enterprise through academic community faculty and staff teamwork. Pathway work has started with one day per week onsite genetic counseling consultations at one local network site. This has now expanded to one day per week of genetic counseling services being available now by telehealth to patients at all community sites. MD geneticist counseling is offered 2 days per month for the community sites after an initial pilot at one community site. Another pilot pathway for genetic assessments is underway with two breast surgeons, trained and supported by the academic CCG team to provide genetic cancer risk assessment (GCRA) to their breast cancer patients with referral to campus for additional genetic counseling or genetics MD for complicated cases as needed. A breast surgeon at another community site is being trained and will provide GCRA services to her patients as well as to other affected and unaffected patients seen by faculty at that site as well as any external referrals. That surgeon also plans to develop a high-risk screening clinic for all cancer types at that community site. Two additional medical geneticists and two additional genetic counselors are being recruited for our second academic campus site in Orange County to provide precision medicine and clinical cancer genomics initiatives.

Standardizing the collection and validated data entry of discrete multigenerational family histories and incorporating these data into individual patients’ cancer or cancer risk data is another project in the planning stages in the value-based framework. It is being worked on in sequential pilots and projects toward enabling our EHR database to capture relevant family and patient histories that can prompt the development of genomics pathways to trigger precision medicine and clinical cancer genomic services as well as personalized therapies within the ClinPath pathway tool. The opportunity to leverage the expertise of the genomics division, working as a team with the community faculty and COH informaticists, holds the promise of developing more pathways for automating risk assessments, genomic counseling, testing, and interpretation as well as clinical trials as part of each patient’s comprehensive care plan. Pathways at COH in other disciplines as described in this manuscript are serving as models to build these to better serve patients across our enterprise.

### 4.8. Expansion of ClinPath Pathways Use for Hematology Diseases

While our acute leukemia teams are working to develop comprehensive care plans and pathways to include diagnosis, therapy, trials, and follow-up for acute myelogenous leukemia (AML) and acute lymphoblastic leukemia (ALL), which is primarily treated on the campus, they have developed referral trees to ease access to campus leukemia experts and their pathways by community faculty and outside providers. For the other major hematology diseases, lymphomas, myeloma, myelodysplastic diseases, and benign idiopathic thrombocytopenic purpura (ITP), which are commonly treated in the community and can involve years of sequenced therapies with growing costs, the ClinPath system has expert hematology disease committees and pathways available in the ClinPath tool alongside the medical and radiation oncology pathways.

The growth of value-based contracting, where payers track metrics on compliance with disease pathways for commonly treated diseases, made adding hematology disease team engagement and enterprise-wide clinical use of the ClinPath hematology pathways a priority for Q4 2020. Since many of these diseases are seen and treated by community faculty, the pathway oversight team reviewed and found that 983 navigations had been done for five types of hematology diseases using the ClinPath tool without any requirement or specific training for their use. While a formal survey was not done, members of the pathway team talked to community faculty and several reported that when they have a question, they use the pathway for hematologic diseases to rapidly inform them of the latest therapies and regimen details for patients with these diseases, especially for the most common diagnoses. They report saving more complex cases for individual campus faculty expert discussions, tumor boards, or second opinion referrals. On the campus side, informal discussion with disease experts raised concerns that clinical trials be more widely disseminated as well as nuanced strategies adopted by campus disease experts who meet weekly to standardize their approaches. While academic faculty welcome calls, referrals, and interactions with the growing community network faculty, they felt that hematology care could be enhanced by hematology pathway adoption.

With the March 2020 California-wide COVID-19 pandemic slowdown in onsite clinical care, the value, staging, and pathway leadership engaged the department chair of hematology in demonstrations of the current EHR, ClinPath pathway tool, and pending interfacing capabilities for the hematologic diseases with clinical trials available in the ClinPath system. Five initial academic and community-based disease leads for diffuse large B-cell lymphoma (DLBCL), chronic lymphocytic leukemia (CLL), Hodgkin’s disease, multiple myeloma, and immune thrombocytopenia (ITP) were identified and those for chronic myelogenous leukemia (CML), myelodysplastic syndromes (MDS), and the other lymphoma subtypes followed. For each disease or subtype of the lymphomas, a video meeting was scheduled, ClinPath pathways and EHR staging data element capture on the current EHR staging forms were shared, and feedback solicited. Disease leads identified additional discrete data elements that track to pathway prompts and a list of those recommended for addition as part of an EHR staging upgrade program was captured and shared with the staging form upgrade build team. Hematology faculty were signed up on the pathway system, made aware of the disease committee meeting timing, and encouraged to participate in adding COH expertise to the pathway regimen discussions. A major drawing point is the ability to add the clinical trials to the flow sheet nodes in the pathway tool as we did for medical and radiation oncology. The disease leads anticipate that this will expand awareness, screening, and ultimately accrual to clinical trials in hematology. The leads have also requested real-time in-basket messaging for patients with specific disease features who would be eligible for a trial and reporting on whether or not a trial was chosen so they could reach out to their colleagues in real time to gain a deeper understanding and to address any barriers to accruals. Having the expanded datasets of disease data and therapy choices will also better inform disease leads on populations served at various sites to better inform opening of targeted clinical trials. After 9 months of meetings, trainings, and engagement, the hematology pathways in ClinPath will be tracked for use and on-pathway compliance starting in December of 2020 with planned ongoing engagement with disease leads and faculty who see hematology patients and build out of more comprehensive analytic reports.

## 5. Oncology Surgery Pathways with ERAS and COH Experience

### 5.1. ERAS Pathways for Value-Based Surgical Care

While pathway work in medical oncology expanded during the early 2000s, surgical pathways, referred to as “early recovery after surgery”, or ERAS pathways were developed out of early work by Professor Henrik Kehlet in Denmark on multidisciplinary care approaches to standardize colorectal cancer surgical care that improved outcomes [51,56]. An international ERAS study group was formed in 2001 to “develop perioperative care and to improve recovery through research, education, audit and implementation of evidence-based practice.” The group later formed an ERAS society in 2010 and a US branch, ERAS@USA in 2016, as data accumulated that orders alone were not enough to improve outcomes but comprehensive approaches to pre, day of, and postoperative (post op) care could improve outcomes in growing numbers of cancer surgeries [80]. The goals of ERAS pathways are to decrease practice variability, lessen morbidity and mortality, and lower costs through education, care coordination, and specific orders before, during, and after cancer surgeries. These are built into care plans on paper or in EHRs to prompt for all agreed upon steps across the surgical journey. Clinical, quality, clinical trial, and financial outcomes can then be analyzed and benchmarked as in other oncology disciplines.

Depending on the surgery, the preoperative (pre-op) processes might include performing an American College of Surgeons developed National Surgical Quality Improvement Program (NSQIP) risk score. This tool predicts the risk of post-op complications “using data from a large number of patients who had a surgical procedure similar to the one the patient may have” [80,81]. This tool is available online and is allowed to be opened from EHRs. Geriatric risk indicators based on data from patients >age 65 were added in 2019 [82]. Using such calculations, for example, post-op nausea and vomiting risks can inform tailored nausea and vomiting prevention education and mediation orders to improve outcomes. The pre-op orders can include breathing training, drain training, smoking cessation, and education on limiting alcohol. They include a review of home medications and any modifications recommended during the pre and postoperative periods. Other goals include improving pain control with limited opioid use through education and standardized orders as well as increasing early mobility to minimize length of stay and enhance the patient experience. The clinical, quality, clinical trial, and financial outcomes of these standardized approaches for specific cancer patients undergoing specific cancer surgeries can then be benchmarked. Teams can use these data for continuous improvement for cancer surgery patients and to better prepare and care for patients at multiple sites. Another key opportunity is standardizing direction of specific high-risk surgeries to high-volume centers where extensive subspecialty surgical expertise has been shown to lower morbidity and mortality and often improve survival across multiple cancer types, systems, and countries despite ongoing controversies on methodologies and the retrospective data reviews [83,84,85,86,87,88,89,90]. Having teams of academic center and community network clinicians working closely together can ease the transitions and the teamwork between academic- and community-based surgical specialists, help to ensure that patients can be easily referred to the academic center for appropriate surgeries per the group pathways while providing continuity and standardization of care using the general and disease-specific ERAS order sets.

City of Hope’s urologic oncologic surgeons started adopting ERAS pathways for cystectomy back in 2008 and garnered a large database on outcome benefits published in 2016 [19]. City of Hope’s academic-based surgeons have the most substantial experience worldwide in performing robotic cystectomy. These patients were an ideal population to design further care improvements around due to the high-risk patient demographic (elderly, many comorbid conditions), complexity of surgery (5–8-h surgeries mixing together multiple organ systems), and challenges of baseline post-surgery recovery (>80% complication rates, >30% major complication rates, and >30% readmission rates). The evolution of a pathway that became the cystectomy ERAS pathway started when the campus urologists began using almivopan to assist with bowel recovery as bowel resection is a key recovery factor in cystectomies. The academic urologists oversaw their high volume of surgeries while integrating teams to deliver standardized care at COH’s academic specialty cancer hospital. They adopted standard orders for pre-op preparation, operative intervention, and post-op management to facilitate recovery for patients. In collaboration with the supportive medicine department, they developed a multidisciplinary rounding team specifically for cystectomy patients that provided an organized plan of care for each patient daily and kept patients actively involved in understanding and participating in their care. Their pathway details the order components for medical management, symptom management, patient education, supportive care, and case management from the operative day through the day of discharge and specifies the follow-up visit order. Adopting a coordinated care program led to several improvements: the hospital length of stay after surgery decreased from 8 days to 6 days, the 30-day complication rate decreased from 68% to 50%, multiple late-stage patients were primarily referred by urology to hospice (in contrast to prior practice where all poor prognosis patients were referred to medical oncology), and some patients and caregivers reported in Press Ganey surveys that they felt much more informed and empowered in their care [19].

### 5.2. Expanding Cystectomy ERAS Pathways to Regional Hospitals and for Prostate Cancer Surgeries

After establishing a successful ERAS program at COH, urology teams rolled out the cystectomy pathway with similar principles to surrounding community hospitals where faculty practiced. Although outcome data show that surgeries of this complexity ideally should be conducted at specialized care centers [59,91], the realities of where our patients reside, their insurance company restrictions, and distributed care delivery necessitate being able to deliver the same standard of care outside COH. Thus, the academic community team faculty partnership built a general ERAS pathway in the order system of the Cerner EHR for a regional hospital where faculty practiced. Over the course of 6 months, they conducted numerous meetings with hospital leadership, nursing leadership, pharmacy, social work, case management, operating room, and nutrition to replicate the model created for the COH campus. At the central academic campus and community sites, teams continue to make iterative implementation improvements using the dedicated pathway functionalities within the EPIC EHR system that incorporates standardized care to reduce variation and cost, organize day-by-day care coordination and documentation, and prompt for discrete capture of outcomes to build robust reporting of outcome and quality measurements.

The urology group next adopted ERAS pathways to drive same day discharges after robotic prostatectomy. Historically, there was a disincentive in fact to discharge these patients the same day as they were considered inpatient only surgeries. However, CMS rule changes have now considered this an outpatient surgery. The group adopted prehabilitation, usage of regional transverse abdominis plane (TAP) anesthesia blocks, early ambulation and feeding, and use of non-narcotic pain medication. This significantly reduced the need for patients to use narcotics, which is beneficial to them individually and in combatting the opioid crisis. Internal data review showed that patients returned home sooner to recover in the comfort of their family and returned to baseline functional and dietary levels much sooner as well.

### 5.3. Expanding ERAS Pathways for Breast Cancer, Colorectal, GYN, Thoracic, and Other Cancer Surgeries

Other surgical oncology teams at COH have worked to develop ERAS pathways for on-campus surgeries as well as for many regional hospitals where our faculty work to achieve similar goals of standardizing surgical care, improving outcomes, and lowering healthcare costs while more actively engaging patients [91,92,93,94]. In one regional hospital, a generalized ERAS pathway was developed and built into their hospital’s EHR to fast-track postoperative mastectomy recovery and minimize narcotic use. At COH, the EPIC EHR has an ERAS breast surgery pathway that incorporates pre-op education, a visit with occupational therapy, and a tailored medication order set. The educational component involves a one-on-one session with a breast team nurse that is performed during the patient’s pre-anesthesia testing visit. Patients receive information on nutrition, exercise, and alcohol/smoking cessation. The information is aimed at preparation for surgery, as well as post-op and long-term recovery strategies. The patients then have an appointment with an occupational therapist to learn about recommended post-op exercises and lymphedema precautions. This “prehabilitation” visit has been found to expedite return to baseline function for patients following breast cancer surgery. Finally, the dedicated breast surgery order set includes medications shown to improve postoperative pain control and minimize intractable postoperative nausea and vomiting [95,96]. The importance of non-narcotic pain control is twofold, as opioids lead to a host of troublesome side effects and COH is focused on combatting the current opioid epidemic [97]. Additionally, the medications given to minimize post-op nausea and vomiting are especially important in breast surgery, as these patients are more likely to develop significant post-op symptoms that prohibit early hospital discharge [98].

The COH ERAS breast surgery pathway can also be tailored to fit an individual surgeon’s preferences or patient needs. The COH breast team has an internal (available on request) detailed mastectomy ERAS pathway order set which details the orders on pre-op education, patient preparation, prehabilitation with physical therapy (PT), or occupational therapy (OT) if needed, educate and order specific medicine prescriptions for post-op care before surgery, and ensures that patients understand the post-op caregiving needs before surgery. On the day of surgery, the anesthesia team is actively involved both at campus and at any of the regional hospitals in their part of optimizing enhanced recovery strategies including as appropriate: intraoperative administration of anti-emetics, intravenous (as opposed to inhalational) anesthetic agents, maintenance of euvolemia, and minimization of opioids. These orders have been aligned with studies showing lessened nausea and vomiting and minimizing post-op opioid requirements, which facilitates faster patient recovery and lessens length of stay [99]. Following surgery, post-op visits, nursing care, and care coordination have helped to lower readmission rates while reducing suffering and promoting faster recoveries. We have surgical specialty teams developing, piloting, or using ERAS pathways in the breast, colorectal, thoracic, and gynecologic surgery programs. As they are developed and implemented with structured orders in the EHR from paper orders, the informatics, analytic, and finance teams are getting engaged to develop outcome reports.

Although expanding comprehensive ERAS pathways to every site our patients are seen in remains in process, other departments at COH and various regional hospitals have reported having benefited from involvement in the ERAS pathway work and order set development. Faculty and staff in the anesthesia department were engaged to align perioperative and intraoperative patient management which, once adopted, were embraced for other surgeries. Surgical faculty have reported informally that some outside anesthesia groups have also eagerly participated in ERAS approaches in our community hospital network to improve the care for patients on and off ERAS pathways.

### 5.4. Challenges with ERAS Pathway Use

While COH surgical specialists uniformly report that they are very successful in getting together and developing clinical agreement and alignment with each other and with other community oncology surgeons at outside hospitals when needed, full implementation of the pathways for all cancer surgeries at COH campus and in all community hospital sites remains a work in progress. A review of the example of the process steps for our mastectomy ERAS pathway is shown in Table 1 and illustrates the complexity of ERAS pathways. To achieve optimal surgical outcomes for cancer surgery patients, ERAS pathways have shown that many different participants in different locations with specific timing, educational, procedural, medication, or care-related tasks need to be facilitated and remain an active focus of our care and quality improvement efforts [92,93,94,95,96,97,98,99]. A model template showing the temporal and detailed types of order considerations for ERAS pathways for cancer surgeries demonstrating this is shown in Table 1.

Our ERAS pathway leaders report an easy process in determining alignment for patient and nursing educational needs and process steps and tracking goals for ERAS pathways among team members. Operationalizing multidisciplinary at home, in clinic, in hospital, and post-hospital steps flawlessly, however, remains challenging. Coordinating the different disciplines with staff internally is slightly easier but made more difficult with the many hospital outpatient and freestanding hospital facilities where our patients receive care. Our surgical lead for the value-based care ERAS development reported that the most challenging aspects of implementation have been related to nursing education, engagement, and data tracking. While there was consensus on the content of both preoperative and postoperative education, identifying the resources and a workflow that could be seamlessly incorporated into each patients’ journey for different cancer surgeries at different sites and in different EHRs outside of our enterprise EPIC system remains a work in progress. With respect to tracking compliance and patients’ clinical outcomes related to the different ERAS pathways, one group plans to pilot a third-party application with an EPIC EHR incorporation. Others are working on various internal EHR tools and the patient portal tools to improve scheduling and prompting of patient education and follow-up care as well as early symptom reporting and triage. In addition to compliance with the pathways, other endpoints being evaluated are length of stay, post-op pain, complications, and survival. Formal measurement of staff satisfaction has not been done but is discussed at surgical department meetings. Patient satisfaction is measured for their overall care by standardized Press Ganey surveys, which consistently report high satisfaction scores but do not evaluate the impact of ERAS pathways alone.

## 6. Supportive Care Pathways

As with other oncology care pathways, those in supportive care have also been shown to be effective in improving care quality and lowering costs based on many trials evaluating care navigation, emotional support for patients and their families with cancer, electronic patient-reported outcomes integrated into care models that address identified symptoms regularly as well as those formalizing goals of care discussions and advanced care planning [20,21,100,101,102,103,104,105].

COH, like many large academic centers, has a palliative care department focused on clinical care and research. Projects have been done in pain management, regular biopsychosocial assessments, and care coordination as well as in end of life care. The larger enterprise focus on value-based care has worked to engage the many expert providers and research teams to more formally identify the projects in supportive care that are essential in the value-based framework to provide interval care, care navigation, and care after cancer with the goals of improving care quality as well as clinical outcomes while optimizing the patient and staff experience. The expansion of several ongoing or supportive care pilots was underway before the COVID-19 pandemic expanded our use of telehealth for patient visits across the enterprise. Support services at COH had discussed pilots to expand social work, nutrition counseling, and other projects or pilots were in discussion to expand supportive care pathways to all enterprise sites. With the launch of telehealth technology enterprise-wide after March 2020 that included an iterative EPIC EHR integration and expansion of telehealth coverage by payers, some of these projects are being expedited and optimized through hybrid in-person and telehealth service pathways. Highlights of some of the pathways in our supportive care programs are:

Expanding Goals of Care Discussions as part of shared decision-making can help to avoid ineffective care, especially at the end of life, and help patients to achieve their broad life goals. While there are numerous other services provided by the Department of Supportive Care Medicine at COH, we will highlight three distinct efforts: the integrated care service, Hospice in the Village, and a goals of care pilot in poor prognosis patients.

The Integrated Care Service is a dedicated supportive care team comprised of a palliative care hospitalist, advance practice provider, social worker, chaplain, and pharmacist who provides intensive interdisciplinary inpatient and outpatient care to patients with multiple complex care needs, including high symptom burden, advanced disease, and challenging psychosocial concerns. In the last year, the team has cared for approximately 300 medical oncology and hematology inpatients, with over 50 being cared for in a primary capacity. As processes and results are studied, they will serve as potential models to deploy in partnering with community hospitals where COH faculty practice to build unique faculty outside hospital and clinician partnerships to achieve pathways for similar care improvements for patients hospitalized outside of the academic campus hospital.

City of Hope’s Hospice in the Village on the Duarte campus has furnished apartment units with full amenities, specially designed for hospice care. Hospice in the Village allows patients to receive hospice care, from their preferred provider, in a private and comfortable home-like environment, when home is not an option. For the last year and a half, overall, 100 patients were cared for in the village, representing over 550 avoided inpatient days. A pilot in a community site for a similar home-based hospice and higher-level care (below acute level) is being explored.

**Figure 7:**
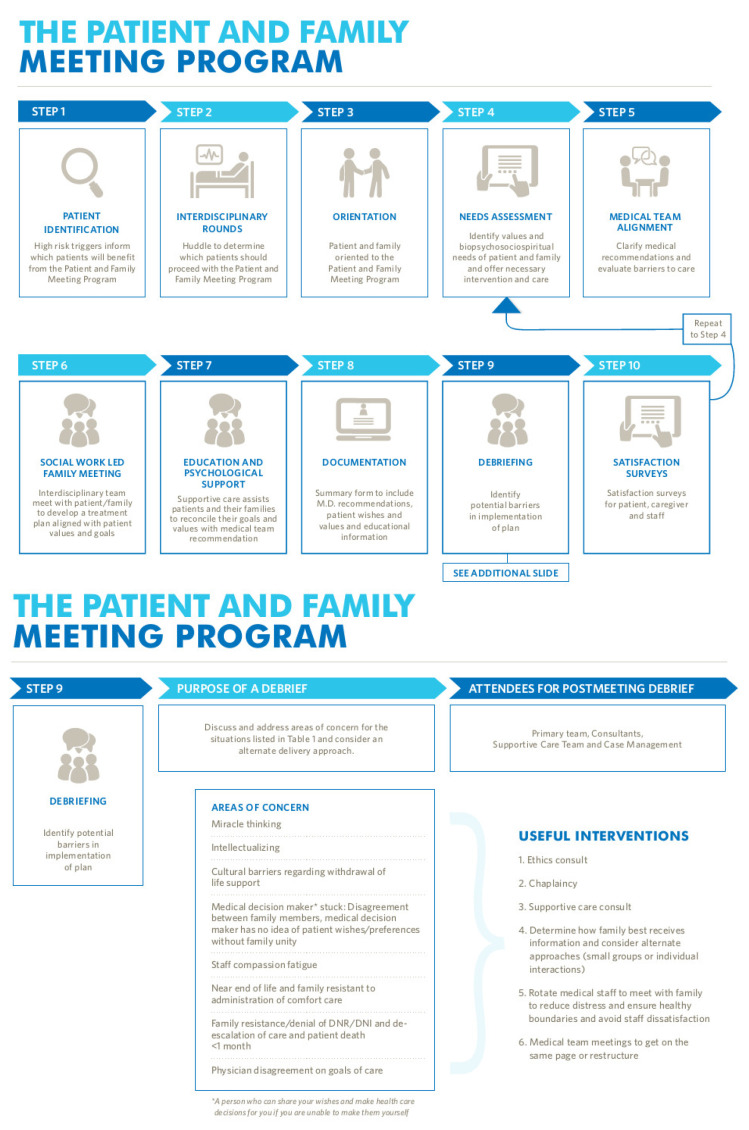
Ten-step supportive care patient and family meeting program pathway with expanded details about step 9 in second section of diagram to improve end of life care at COH.

A collaborative hematology, oncology, and supportive care pilot is underway utilizing various means to identify high-risk patients, including supervised machine learning, which triggers a supportive care pathway that includes patient screening (including assessing patient perception of prognosis), advance care planning, training on communication, structured goals of care documentation, and engages supportive care disciplines based on patient and family identified needs. The structure of the patient and family meeting pathway is shown in Figure 7, with further information available at the COH website [106].

Implementing Advanced Care Planning Pathways across the enterprise is another key component of our value project’s care after cancer pillar. Advance care planning is often poorly incorporated in oncology practices, with reported advance directive (AD) rates generally less than 50% [107]. Concerted efforts were made to improve the overall number of ADs in new patients across the enterprise and specifically for patients undergoing hematopoietic stem cell transplantation (HSCT). The Department of Supportive Care Medicine at COH, in collaboration with medical faculty and administrative support, created a patient-centered ACP pathway program.

The first two years (2013 and 2014) broadly focused on all new COH patients. The last two years (2015 and 2016) included a specific focus on patients undergoing HSCT. The primary goal was a completed AD in the electronic medical record before day 0 of transplant. In addition to provider and transplant team engagement, major time points for supportive care integration to facilitate AD completion were identified, including: (1) registration, (2) new patient orientation, (3) the clinical visit when transplant was decided, (4) pre-transplant education class, (5) clinical social work psychosocial assessment visit, and (6) the pre-transplant hospital days. Between 2012 and 2016 at COH, 1784 transplants were performed. For HSCT patients in 2012, baseline AD capture rate before day 0 of transplant was 28.6%. With the institutional AD program, the AD capture rate before day 0 of transplant was 31.6% for 2014, compared with 2012 (odds ratio, 1.17(95% CI, 0.85–1.60); *p* = 0.33).

With both institutional and hematology-specific programs, the AD capture rate before day 0 was 69.5% for 2016, compared to 2014 (odds ratio, 4.30 (95% CI, 3.14–5.91); *p* < 0.001). While the institutional AD program in 2014 insignificantly impacted HSCT AD completion rates, improving the rate of AD completion from 28.6% to 69.5% in HSCT patients required both institutional AD efforts and a targeted pathway program [108].

After this progress, the team’s advance care planning efforts returned to focus on building a scalable advance care planning pathway to support the advanced care planning and AD completions in our 31 network clinics. While advance directives in California can be finalized by two witnesses or a notary, at COH, for our predominantly cancer-focused population, we have opted to not solicit witnesses and elected to offer complimentary notarization services for healthcare documents to minimize a major identified barrier to AD completion. Presently, a pilot pathway is under way, leveraging the Prepare for Your Care advance directive developed by the University of California San Francisco (UCSF) and having these advance directives notarized electronically. Several staff advance directives have been successfully completed and initial patient tests are under way to ensure ease of use, feasibility, and adoption. Building the structured steps and documents into the EHR is on the project roadmap so the AD pathway tools can be available enterprise-wide and outcomes can be tracked and reported for targeted improvements. Additional information regarding City of Hope’s advance care planning program is available on the website [109].

Pathways to address, prevent, and treat cancer symptoms through standardizing assessments, triage, and treatments is not only a key component of our value framework’s management of patients on therapy pillar, it is a key component of ASCO’s Quality Oncology Practice Initiative (QOPI) metrics and Center for Medicare and Medicaid Services’ (CMS) Merit-Based Incentive Payment System (MIPS) measures, which we track for compliance [110,111]. Standardized use of patient reported outcome tools has also been shown to improve survival as well as relieve suffering [20,112,113].

Phone Triage Pathways: Our EHR regimens are all built to include evidence-based nausea, vomiting, and hypersensitivity medications that can then be obtained pretreatment and discussed at the chemotherapy teaching visit. The medications are based on the NCCN low, minimal, moderate, and high emetogenic risk of the regimen, which automates a major quality metric to ensure appropriate pre- and post-medications.

Support Screen Pathways: Our management of care pillar for value-based care also uses pathways for phone triage, biopsychosocial, and per visit symptom assessments and care. We implemented the triage pathway as a pilot in 2018 with the campus breast disease team, which was expanded to campus medical oncology clinics in 2019 and is now being prepared for piloting in the community sites using the EPIC phone triage tool with embedded Schmidt Thompson evidence-based assessment pathways and per disease team or site standard operating protocols for triaging patients with cancer symptoms. We are expanding the nationally recognized work of the supportive care faculty’s biopsychosocial Support Screen tool with validated questionnaires and integrated support materials or referrals in a pilot at two community sites. Patients, at specific visits or remotely, can respond to questions on iPads in clinic or through our EPIC MyChart portal. Ongoing work that will be informed by the community pilots will work to address the challenges of addressing complex biopsychosocial needs through hybrid models using community resources supplemented by academic experts. Challenges of identifying resources and activating them based on individual patient needs across an enterprise remains an active project to expand these well validated supportive care pathways for biopsychosocial symptom monitoring and interventions across the enterprise.

**Figure 8:**
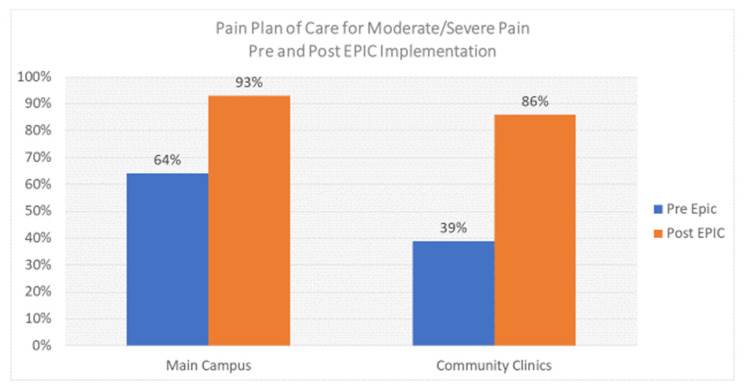
Pain plan of care compliance for those reporting moderate to severe pain. Blue represents results from the 3 QOPI reporting quarters (Fall 2016, Spring and Fall 2017) from the previous EHRs (Allscripts on campus and TouchWorks in community sites) prior to the EPIC implementation. Orange represents results from the 4 QOPI reporting quarters (Spring and Fall 2018 and Spring and Fall 2019) after the December 2017 EPIC transition for both the campus and community sites.

**Figure 9:**
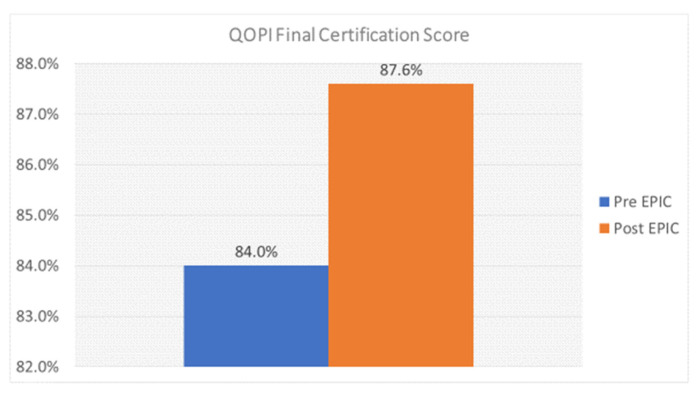
COH enterprise QOPI final certification scores. Blue represents the 3 reporting quarters (Fall 2016, Spring 2017 and Fall 2017) before the -EPIC implementation. Orange represents the 4 QOPI reporting quarters (Spring and Fall 2018 and Spring and Fall 2019) after the EPIC go live December 2017.

Oncology Review of System Pathways: We have also built a multidisciplinary community developed oncology review of symptoms (ONC-ROS) questionnaire into a patient-reported outcome questionnaire available to patients through the EPIC MyChart portal before each visit or in clinic within the EPIC rooming questionnaire that medical assistants can complete with patients prior to visits. The 39 questions cover the most common toxicities for cancer patients receiving medical oncology, surgical oncology, and radiation oncology therapies. Community doctors preferred one questionnaire that medical assistants, who help multiple doctors, could be trained and become familiar with for efficient use. The 39 symptoms are divided into systems which require answering only the positive symptoms; then, with one click, the remaining symptoms can be noted as negative. For the nine symptoms that are monitored for the ASCO-QOPI and CMS-MIPS metrics (n/v, diarrhea, constipation, anxiety, depression, pain, fatigue, falls, and shortness of breath), a positive response opens additional questions to assess patient acceptance of that toxicity or desire for interventions that can be addressed at the visit. The questionnaire responses integrate into the EPIC visit note template as the review of systems for clinician review and editing to ensure symptoms are addressed, which has been shown to lessen hospital and ER visits while speeding up complex documentation to support appropriate billing levels and providing mineable data [20]. The use of the ONC ROS after teachings on capturing a pain score and pain plan of care in EPIC after the December 2017 go live numerically improved the capture of the pain plan of care QOPI metric average or the three reporting periods (Fall 2016, Spring and Fall 2017) before our EPIC transition compared to the four reporting periods (Spring and Fall 2018 and Spring and Fall 2019) after the EPIC transition. This occurred in both the campus (64% pre-EPIC to 93% post) and community sites (39% pre-EPIC to 86% post) as shown in Figure 8. COH’s quality team, who collect and report the QOPI scores from the EHR, reported that increased symptom-reporting data from use of the ONC ROS questionnaire in patient visit notes as well as having the NCCN nausea risk level medication orders built into the chemotherapy orders in our EPIC EHR were the main reasons for our improved enterprise QOPI scores for the reporting periods after EPIC compared to those before EPIC implementation. As shown in Figure 9, the score improved from 84% pre-EPIC without the OncROS and nausea regimens built into regimens to 87.6% post-EPIC with the use of the OncROS and nausea medications built into chemotherapy regimens for the enterprise. Ongoing work evaluating which patient-reported outcome tool might work with our triage pathways, multidisciplinary pathways, the EPIC EHR, and our diverse patients is in progress toward a standardized PRO tool for all services. Our criteria for choosing an enterprise-wide tool include patient and provider satisfaction for ease of use, efficacy to improve clinical care, and integration of the PRO tool into care pathways to improve value-based outcomes and ease documentation to support team-based triage communication and provide mineable data to support quality assessments.

## 7. Other Enterprise Academic Community Teamwork Supporting Personalized Pathways for Value-Based Care Delivery

Disease team meetings include academic and community doctors and APPs who get to know and develop respect for each other. They discuss the newest science, clinical trials, and care opportunities that are not yet in pathways, pending disease committee reviews and the 6–12-month processes. Disease teams maintain internal pathway flow diagrams with some modifications embraced before they are formally available in national pathway tools.

New pre-ClinPath pathway update tool for practice changing therapies in development: COH has identified the need for a new tool to capture and share in a standardized monthly update format new practice changing updates agreed to by disease teams. Tracking analytics are being developed concurrently to track the sharing of new practice changing knowledge with the agreement to add to pathways, the availability in the pathway tool, and the uptake by faculty across the enterprise for long-term quality improvement work. Baseline studies in three tumor types are underway to inform this new process.

Tumor boards: These are all now done for tumor types by teleconferencing so the multidisciplinary teams of campus and community faculty actively participate. Some community doctors report that they are submitting at least one case to each tumor board and regularly submit to the tumor board of 1–2 cancers which they may be particularly focused on. They report that the move to tele-tumor boards for all faculty has increased participation and feel that they are sharing and learning more from the entire multidisciplinary team in very open and collegial discussions. This is true for the weekly molecular tumor board as well.

Joint clinics: Many academic faculty have agreed to cover or see patients from time to time in different community clinics and some community doctors spend regular time in academic campus clinics. The academic faculty appreciates the tight knit and efficient teamwork at community sites who evolved to be very efficient in a highly managed care world. Academic experts get to share one-on-one the latest molecular or other discoveries during real-time patient encounters with their community faculty colleagues and observe the use of ClinPath and other pathways at community sites. Friendships and increased collegiality have blossomed, which encourages ongoing sharing that offers every patient access to cutting-edge knowledge. A system to facilitate second opinions between the campus and community faculty has also helped to increase patient peace of mind while allowing them to receive care at the site closest to their home.

Regional, disease team and campus symposia and lectures are shared with all faculty. The medical oncology department holds quarterly half-day symposia where campus and community faculty present the latest science and care pathways as well as sharing clinical trial, value-based care, and programmatic updates. Disease teams also hold retreats to share the latest disease-specific knowledge and clinical trials, which is another helpful format for networking and building of collegial relationships. The pandemic restrictions on in person meetings led the medical oncology department to hold their sixth symposia in October of 2020, where 118 members participated in a 5-h update on science and clinical programs. The feedback was enthusiastic for the format, where there was active engagement and extensive group and interpersonal sharing of ideas in the chat function while members avoided having to travel from family on a weekend.

## 8. Other Value-Based Care Framework Projects Integrated with the Pathways Programs

Development of enterprise disease trees is a newer initiative being done to aid further in the standardization process of developing personalized multidisciplinary care plans throughout the enterprise. Community disease and regional leaders are closely involved with academic-based colleagues in building the initial decision tree for breast cancer. Next up in the queue are GI and GU multispecialty decision tree builds. The campus disease trees include locations for complex surgeries or other cancers as well as timing and genomic testing for both somatic and germline mutations. Within these disease trees is an enterprise-wide initiative to extend germline and somatic genomic testing to all patients as well as the integration of the results into the disease pathways. This will be piloted in one site (South Pasadena) close to the academic center and then expand to all sites by 2021. Pulling in the disease-specific pathways from medical oncology, hematology, radiation oncology, and surgical oncology, along with supportive care pathways, nutrition, genetic counseling, and testing as well as other rehabilitative services, will help to define when in a patient’s course of care is provided and in which sites by which experts.

Increased interoperability of EHRs, especially EPIC, is an ongoing project. An advantage of EPIC, which, in CA, is the primary EHR for major university cancer centers (COH and Stanford) as well as the UC system hospitals (UCLA, UCI, UCSD, UC Merced, UCR, and UCSF), as well as the Kaiser Permanente Health System, which serves 40% of California residents, is the ability to review past records, therapies, and diagnoses, which can better inform current care plan development. Continued regulatory pressure to improve the interoperability of all EHR systems and the laboratory, imaging, and diagnostic companies is important to ensure that accurate patient data are available to treating clinicians to facilitate proper care plan development.

Improved functionality of EHRs to capture data more efficiently and support customized care planning is an ongoing focus for most oncologists to empower value-based care initiatives. The goal that we started with at COH, to develop personalized multidisciplinary CARE PLANS for each patient, continues to advance as disease and discipline-specific pathways are implemented, which can be then be made for each patient with our fast and frugal tree approach (described above). Getting to the point where every patient can be given a comprehensive, multidisciplinary care plan that can be navigated and managed remains a top priority for COH and other organizations focused on actualizing all the elements of VBCC.

Staging initiative for discrete data elements engages faculty in defining the data elements needed for decision support and downstream outcomes for each type of cancer. The AJCC has identified components for staging diseases that now include three categories: traditional staging elements, prognostic features, and clinical care features [114]. While the latest, AJCC 8, which became active in the US for staging in January of 2018, expanded these ideas in breast and some other cancers, most cancers do not have these details defined by the AJCC [114]. These are the growing number of discrete disease and patient-specific data components that track to pathway prompting when the patient goals and other comorbidities make it likely that a standard evidence-based pathway will have the best outcome [115]. Capture of these discrete elements facilitates the interfacing of data entry in the main EHR for an organization, then linking those features into decision support tools to minimize duplicate data entry and improve practice efficiencies, as we have pioneered and described above. Our initial 10-month, enterprise-wide 2020 staging initiative showed that 92% of all new patients seen in medical surgical and radiation oncology between January and September 15 of 2020 had required staging data elements entered in the EHR. The next steps for 2021 will be expanding the required data elements for entry and dashboard reporting to clinicians to include elements that are capturable in the EHR that can map to decision support in the pathway tool while we simultaneously expand the tumor types we have full mapping of EHR data elements into the pathway tool and back to therapy orders for medical and radiation oncology, as discussed above.

Oral hemotherapy drugs are built into the EHR as regimens: In preparation for further integration between our EHR and the ClinPath pathway tool, we identified 58 oral cancer drugs which had not been built into our chemotherapy ordering system (Beacon for EPIC). These are included in 96 protocols which will be prompted from the pathway program, so both academic and community medical oncologists then hematologists were recruited, agreed to build specific protocols including the references, associated NCCN nausea level, dosing, visit, lab, and specific drug guidance using a standardized Excel tool. Examples of current protocols were shared at individual or small group teaching sessions held through video visits. The builds for all but the last 25 protocols have been completed, including validation by an oncology Pharm D since April 2020, which was stimulated by the March 2020 COVID-19 pandemic work from home emphasis. We anticipate that the remaining 25 regimens will be built and validated by December of 2020 and have a goal that all of the 96 regimens will be available in the EPIC Beacon ordering system by Q1 2021. Once completed, we will launch a best practice alert if a clinician orders an oral chemotherapy agent in the stand-alone prescription system of the HER rather than ordering it in the fully integrated chemotherapy order section in the EPIC Beacon system. The advantage of the build in the specific chemotherapy order system is the tie into the pathway, prompting the ability to standardize chemotherapy teaching visits in each order, support for the authorization processes, and integration into our recently launched specialty pharmacy program. With these integrations, any additional coverage needs for high-cost specialty and other cancer therapies can be identified and addressed early on while also facilitating analytic reporting on specific patient tumor types and disease features treated by specific regimens over measurable time periods for clinical and financial outcomes.

Outreach to community hospitals and ambulatory surgery centers is ongoing to share ERAS and other supportive care pathway work and engage diverse teams in meeting the care steps. Working across systems remains a challenge as noted. There is a clear focus, however, on developing these so that, wherever the patient chooses to get their care, the same high-quality care standards will be incorporated into the care processes and embraced by all staff, whether they work for our enterprise, a partnered entity, or a standalone facility. An advantage of our academic community faculty team model is the easy sharing and customization abilities of our clinicians and administrators so that the additional tools and processes developed at our NCI designated comprehensive cancer center can be shared with community-based hospitals and clinicians outside our enterprise.

**Figure 10:**
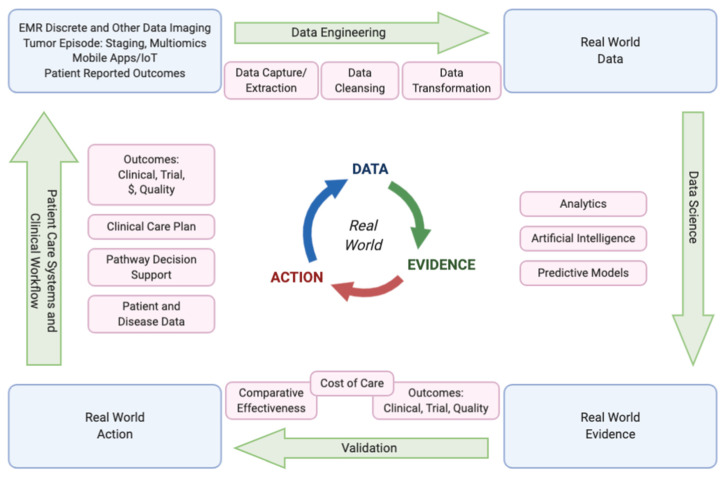
City of Hope’s overriding digital framework to empower value-based care and real-world insights.

The COH Enterprise data warehouse collects data elements from all digital platforms used by the enterprise and supports VBCC as well as research and other initiatives. It is overseen by a governance committee with clinical informatics, precision medicine, and information technology experts, who make data available to empower care and insights. From the data warehouse, Tableau dashboards can be created for feedback to clinicians and administrators along with many other informatic formats to process large datasets for study, some of which are shown as figures in this manuscript. The digital framework to turn information into real-world data is described in Figure 10.

## 9. Challenges of Keeping Pathways Updated and Minimizing Burnout

Among the many workstreams we have discussed here, two other challenges should be highlighted: the challenge to keep decision support tools up to date in real time and the importance of engaging physicians in the design and efficient implementation of value-based projects as team members to minimize burnout. Our enterprise has recognized and is addressing methods to keep pathway tools up to date and engage clinicians in the many value-based project processes. These challenges are both minimized by the integrated academic community faculty foundation teamwork, which not only gains from the diverse experiences and wisdom of the faculty but can help to reduce the growing burnout rates and earlier retirement events for cancer clinicians. Three components of burnout described by the AMA Steps Forward highlight a culture of wellness, personal resilience, and practice efficiencies as the major areas contributing to burnout [116]. Internal COH faculty surveys identified practice efficiencies as the most important factor contributing to burnout in our faculty as collected and shared at regular faculty meetings. These surveys have prioritized work to address efficiencies and teamwork as we implement VBCC projects with academic and community team inputs.

Implementing multidisciplinary pathways across an enterprise and expanding to outside organizations faces the ongoing challenge of keeping pathway and decision support tools up to date in real time so that clinicians can deliver value-based care across the enterprise. Oncology is fortunate to have so many continuing new discoveries that can improve outcomes, but they require real-time tools and programming to bring practicing changing diagnostic and therapeutic knowledge to clinicians. Doing this with academic disease team leads working with integrated academic community disease teams helps to share the knowledge and build real-time enterprise tool updates until the larger pathway decision tools are updated. This can also improve workflows that can enhance patient and practitioner satisfaction [116]. It can also help to reduce burnout from extended work hours from individual clinicians having to individually evaluate the copious amounts of detailed new cancer information for the many kinds of cancer patients that they see.

A second challenge is to engage clinicians and staff teams in pathways and value-based initiative work so that the data entry, programming of digital tools, and workflow processes align to minimize their contribution to burnout while empowering evidence-based care delivery. Faculty who are encouraged and rewarded by leadership to participate in their passion for cutting-edge care and clinical research, while contributing to enterprise and professional initiatives, can lessen the sense of loss of meaning in work and a feeling they are a replaceable robot in an assembly line of patient care. Dashboard feedback that engages doctors and their teams with the results of discrete data entry and clinical trial outcomes can fuel pride in their and their care team’s work with patients as well as in their role in the success of the larger enterprise. While many clinicians expressed initial push back on the increased workloads to enter discrete staging and pathway navigation data, as familiarity with the systems increased and the integration of the tools as well as the clinical trials expanded, there was a growing sense, which has not been formally measured, that the time spent entering defined, discrete data once is likely offset by time savings from authorization clerks, searching for patient information to inform clinical trial prioritization, expanded efficiencies in database-related clinical research, improved inter-specialty communication of the patient’s disease and course, and, as sequences of disease descriptions build across a patient journey, markedly speeding up the ability of clinicians to gain a rapid understanding of what a patient has had, what they were treated with, and where the next steps in diagnostics, follow-up, or subsequent therapies should be. All of these tradeoffs would benefit from formal study that might also help to inform new pilots about return on investments regarding who and when on the team with what training are optimal for specific data entry if not the doctors or APPs [116].

This work can also be informed by many experts outside of medicine as we continue to iterate and learn from pioneering efforts. Dr. Etienne Wenger, PhD is one such globally recognized thought leader in the field of learning theory and its application to business who is a pioneer of “community of practice” research. Especially relevant to our work is his statement that “Communities of practice are important to the functioning of any organization, but they become crucial to those that recognize knowledge as a key asset” [117].

COH as an enterprise is addressing these two challenges along with the many others discussed. The pathways and care plan development programs benefit with leadership supporting active clinician engagement across our academic and community cancer center sites, which addresses several major components to minimize burnout: improving work place efficiencies, reducing social isolation at work, and the sense of loss of meaning in work that care standardization without engagement can bring. This has helped clinicians and staff to embrace the shift to value-based care through personalized precision medicine with integrated clinical research. The addition of teamwork across the enterprise by clinicians, managers, nurses, pharmacists, administrators, quality and informatics experts with our IT teams also iterates improvements in EHR functionalities, allowing more patient-reported input and team-based data entry, which can free up clinician time to analyze data and engage patients in developing a personalized, multidisciplinary care plan. The many projects outlined with multidisciplinary oncology pathways as a key component towards enterprise delivery of high-quality cancer care can be done with thoughtful attention to methodologies and team engagement so that value-based care, defined as the combination of high-quality care, patient satisfaction, provider satisfaction, and enterprise health at the appropriate costs, can be achieved.

## 10. Conclusions

Patients, clinicians, patients’ families, employers, and payers want to have the best care plan available, discussed and implemented seamlessly with ongoing patient support to achieve the best cancer care outcome, whether this is a cure or improved quality of life. Diagnostic testing, genomics, imaging, and clinical assessments have increasingly complex discrete data elements that need to be transformed into actionable information and decision support. This knowledge also needs to be communicated effectively in the shared decision-making process with patients and payers. Pathway programs with decision support tools integrated into the clinical workflows can inform high-quality clinical trial, treatment, and supportive care decisions efficiently when overseen and supplemented by disease teams informed by integrated academic community faculty partnerships. This knowledge, integrated into EHRs and digital tools, can empower true VBCC throughout a fully integrated, multisite enterprise.

Thus, while many see oncology pathways as focusing only on medical oncology and hematology regimens, they are just as important for specific patient populations such as geriatrics, clinical cancer genetics, AYA, and pediatric populations as well as for surgical oncology, radiation oncology, and supportive care. Combining pathways personalized to each patient can then build the evidence-based comprehensive care plans to optimize each patient’s cancer journey for their best health outcome. Foundational work on multidisciplinary oncology pathways, which started with COH’s early engagement and leadership with the NCCN, has progressed to the development and implementation of disease-specific, supportive care, subpopulation-specific (i.e., geriatrics), and multidisciplinary pathways toward our goal of comprehensive, individual patient care plans. This work, coordinated under our Value Realization enterprise project, is overseen by faculty and administrative teams with diverse scientific and practical operational knowledge as well as academic and community expertise. The partnered teams have facilitated this pioneering work as an enterprise to develop, implement, and analyze the methodology and infrastructure to care for each cancer patient with a personalized care plan based on their cancer diagnosis, detailed and evolving molecular diagnostics, clinical trials, and bio-psychosocial needs with tools to support engagement in shared decision-making at initial diagnosis and any subsequent recurrences.

As more community oncology practitioners with practical implementation and local cultural expertise join local, regional, and national networks with experts who maintain the intense, detailed focus in subspecialty areas of oncology care, this combined knowledge and perspective can be shared through standardized decision support oncology pathway tools supplemented with real-time, team-led modifications as new knowledge helps us to achieve better outcomes with less toxicity for patients, regardless of where they live. Our COH enterprise commitment to one standard of high-quality care for every patient led to the pilots and multidisciplinary oncology pathway programs using tools, teams, and processes that can serve to inform others as network affiliations grow. While this work is empowered by the team-based, one faculty, one standard of care philosophy across our enterprise, there are many operational and implementation steps to deliver and measure this care standard. The work has benefitted from the combined strength of the academic faculty’s deep scientific knowledge and focus with the community faculty’s deep clinical and operational knowledge in partnership with administrators, project leads, informaticists, IT experts, and committees.

Implementing and integrating multidisciplinary oncology pathway programs also benefits from other partnered projects with subspecialty groups, IT interfaces, EHR functionalities, care teams, and analytic reporting as reviewed. While multidisciplinary oncology pathways are key components in the delivery of high-quality cancer care, they are only one of the many important component projects of VBCC. Achieving measurable delivery of all component projects within the three VBCC pillars of evidenced-based care, care management, and care after cancer with high levels of patient satisfaction, clinician satisfaction, and institutional health with optimized costs remains a work in progress. Early reports of COH’s successes and the successes reported by others can serve to inform and inspire ongoing work in VBCC [8,11,26,27,30,34].

## Figures and Tables

**Table 1:** Detailed timing and order categories to address in ERAS pathways cancer surgeries.

Pre-Operative Orders Weeks Ahead Addressing:	Intra-Operative Orders Addressing:
Education visit and potential handouts	Fluid management
Assess post-op n/v risks and educate	IV/inhalational anesthetics
NSQIP surgery risk calculator	Regional anesthesia
Smoking and alcohol cessation	Deep Venus Thrombosis prophylaxis
Breathing exercise teaching	
Diet and activity guidance	**Post-Anesthesia Care Unit:**
Prehabilitation OT/PT teaching	Pain guidelines
Lab orders	Catheter guidelines
Medication education for pre-op and post-op	
Bathing instructions	**Extended Stay Orders:**
	Nursing care
**Pre-Operative Orders Days or Day Before Surgery:**	Medications
Patient instructions prior to surgery	DVT prophylaxis
Nasal and skin disinfection	As needed medications
Medication fills for post-op needs	
Prehabilitation OT/PT teaching	**Post-Op Orders Addressing:**
Pre-anesthesia testing	Follow-up nurse triage call
Pre-op fasting	Post-op visit scheduling
Pre-op medications	
Pre-op DVT prophylaxis	

## Data Availability

Data sharing is not applicable. Data was collected for quality improvement, is proprietary thus not publicly available.
